# Neuropsychiatric Adverse Events During 12 Months of Treatment With Efavirenz in Treatment-Naïve HIV-Infected Patients in China: A Prospective Cohort Study

**DOI:** 10.3389/fpsyt.2021.579448

**Published:** 2021-02-24

**Authors:** Wei Hua, Sen Wang, Xi Wang, Ying Shao, Yali Wang, Jiangzhu Ye, Bin Su, Taiyi Jiang, Tong Zhang, Hao Wu, An Liu, Qunhui Li, Supriya D. Mahajan, Zaicun Li, Lijun Sun, Lili Dai

**Affiliations:** ^1^Center for Infectious Diseases, Beijing Youan Hospital, Capital Medical University, Beijing, China; ^2^Department of Infectious Diseases, Institute of Infectious Diseases, Huashan Hospital, Fudan University, Shanghai, China; ^3^Division of Allergy, Immunology and Rheumatology, Department of Medicine, Clinical & Translational Research Center, University at Buffalo, Buffalo, NY, United States

**Keywords:** efavirenz, antiretroviral therapy, anxiety, depression, sleep disturbances

## Abstract

**Background:** Efavirenz (EFV) is widely used in antiretroviral therapy (ART), but the incidence and risk factors of neuropsychiatric adverse events (NPAEs) after EFV treatment have rarely been studied in Chinese ART naïve patients.

**Methods:** This prospective cohort study assessed HIV-infected patients initiating antiretroviral treatment with EFV to determine prevalence of and factors associated with NPAEs over a 12-month follow-up period using the Hospital Anxiety and Depression Scale (HADS) and the Pittsburgh Sleep Quality Index (PSQI).

**Results:** A total of 546 patients were enrolled. Prevalence of anxiety, depression, and sleep disturbances at baseline were 30.4, 22.7, and 68.1%, respectively. Six patients discontinued treatment due to drug related NPAEs. Treatment was associated with improvements in HADS-A, HADS-D, and PSQI scores over the 12-month follow-up, and the frequencies of patients with anxiety, depression, and sleep disturbances significantly decreased after 12 months. Abnormal baseline HADS-A, HADS-D, and PSQI scores and other factors, including high school education or lower income, unemployment, divorce, and WHO III/IV stages, were associated with severe neuropsychiatric disorders over the 12 months.

**Conclusions:** These findings suggested EFV discontinuation due to NAPEs was low, and the HADS-A, HADS-D, and PSQI scores after 12 months of EFV treatment were associated with several risk factors. The clinicians should keep in mind and routinely screen for the risk factors associated with neuropsychiatric disorders in HIV-infected patients.

## Introduction

Efavirenz (EFV) is a non-nucleoside reverse-transcriptase inhibitor (NNRTI), which is widely used for the treatment of human immunodeficiency virus (HIV) infection in antiretroviral therapy (ART)-naïve patients. The clinical use of EFV was first approved in 1998, and the 2016 World Health Organization (WHO) treatment guidelines recommended EFV be used in combination with tenofovir and lamivudine or emtricitabine as the preferred first-line ART regimen in low- and middle-income countries (1). The clinical efficacy of EFV has been proven by several studies, supporting the idea that starting therapy with EFV induces a better virologic response, and is less frequently associated with severe adverse events than starting therapies with other regimens containing NNRTIs or protease inhibitors (PI) (2–5). Once-daily dosing and its relatively low cost also support the use of EFV as the preferred option for initiating ART, particularly in low-income settings.

Although EFV is highly efficacious in reducing HIV viral loads, its clinical use is reported to be associated with neuropsychiatric adverse events (NPAEs), including confusion, dizziness, nightmares, sleep disturbances, anxiety, and depression, as well as more severe symptoms including suicidal ideation and psychosis (6, 7). The incidence of NPAEs upon initiation of treatment with EFV-containing regimens has been reported in 25–40% of HIV patients (8–10). EFV-related NPAEs occur, most commonly, within the first few days of treatment, but the incidence tends to decrease after the first month of treatment (10). In some cases, however, these adverse events can persist for months or may not resolve at all (11). The cellular and molecular mechanisms responsible for EFV-induced neurotoxicity are not entirely known, though EFV induces autophagy and mitochondrial inhibition in neurons, which may lead to the central nervous system (CNS) toxicity associated with NPAEs (12, 13). Although there are concerns about the risk of EFV-related NPAEs, a systematic review found the relative risk of discontinuation due to adverse events was higher for EFV compared to other first-line options, but the absolute differences were <5%, with no reported suicides (14).

However, EFV remains one of the most widely used ART drugs in China, which has a large population of the global burden of patients with HIV infection, partly because of limited drug accessibility and lower NNRTI transmitted drug resistance. The use of EFV has been recommended and supported under the “Four Frees, One Care” policy in China. The EFV-related NPAEs and their impact on drug adherence and quality of life still require further study; to the best of our knowledge, there is no relevant literature reporting on these NPAEs in Chinese populations. Recently, some studies reported that a once-daily, reduced dose of EFV (400 mg) was associated with a lower risk of NPAEs and fewer patients stopping treatment, with no negative impact on the drug's treatment efficacy (15, 16). A clinical trial in China also demonstrated a regimen composed of EFV 400 mg was non-inferior to the standard dose regimen but with a lower frequency of adverse events (17). However, whether the reduced dose need to be widely and urgently promoted among Chinese HIV/AIDS patients still need to discuss. Additionally, since the recommendations for rapid ART were proposed by the WHO, which supported the initiation of ART as soon as possible after confirming HIV infection, the time interval from diagnosis to the initiation of ART has been significantly shortened. It is unknown, however, what impact early treatment with EFV has on the risk of associated NPAEs.

To address these knowledge gaps, the aims of this study were to evaluate EFV-related NPAEs including anxiety, depression, and sleep disturbances over a 12 month follow-up period in Chinese patients with HIV initiating a first-line regimen containing EFV (600 mg, once daily), and to prospectively identify risk factors associated with severe NPAEs during EFV treatment. The results of this study could help clinicians gain better experience in managing NPAEs of EFV treatment.

## Methods

### Study Design

In this prospective observational study, we recruited HIV-positive patients who received care at Youan Hospital in Beijing from July 2014 to April 2015 and were followed up for 12 months after the initiation of treatment. Youan Hospital is one of the most important HIV/AIDS health care centers in China, where more than 10,000 HIV-infected patients on ART are followed regularly. The inclusion criteria included: (1) ART treatment-naive patients diagnosed with HIV infection; (2) at least 18 years of age; (3) had not been pregnant within the prior 3 months; and (4) initiated treatment with EFV (600 mg/day) + lamivudine (3TC) + tenofovir (TDF) or zidovudine (AZT) + lamivudine (3TC). Exclusion criteria included the presence of any condition that could affect the ability to complete the study questionnaires. Patients diagnosed with an ongoing psychiatric illness and those receiving pharmacological treatment for psychiatric problems were also excluded. The prescription of EFV was determined solely by the patients' physicians according to established guidelines, with treatment administered to the patients during routine clinical practice at the hospital after enrolment.

The study was approved by the Clinical Research Ethics Committee of the Beijing Youan Hospital (LL-2019-038-K) in accordance with the tenets of the Declaration of Helsinki. All subjects provided written informed consent.

### Data Collection

The primary data were collected at baseline (M0), at 2 weeks (M0.5), and 1 (M1), 3 (M3), 6 (M6), 9 (M9), and 12 months (M12) after initiation of treatment. At each time point, depression, anxiety, and sleep quality were assessed using standardized, validated self-reported measures. Sleep disturbance was evaluated using the Pittsburgh Sleep Quality Index (PSQI), and symptoms of anxiety/depression were assessed using the Hospital Anxiety and Depression Scale (HADS).

Basic demographic characteristics and clinical data were collected systematically at each timepoint by reviewing the medical records. These factors included age, sex, race, date of HIV diagnosis, stage of HIV infection based on WHO classifications, viral load, cluster of differentiation 4 (CD4) counts, and psychiatric history.

### Evaluation of Sleep Quality

Sleep quality and disturbances were assessed using the Chinese version of the PSQI, a self-rated 19-item questionnaire that scores seven sleep components: subjective sleep quality, sleep latency, sleep duration, habitual sleep efficiency, sleep disturbances, use of sleeping medication, and daytime dysfunction. The sum of the scores of each of these seven components yields one global score ranging from 0 to 21, with higher scores indicating poorer sleep quality; a score between five and ten indicates mild sleep disturbance, and scores over 10 indicate severe sleep disturbance (18, 19). The PSQI has been validated in the Chinese population (20).

### Evaluation of Anxiety and Depression

Anxiety and depression were assessed using the Hospital Anxiety and Depression scale (HADS) developed by Zigmond and Snaith (21). It comprises 14 items, seven of which relate to symptoms of anxiety (HADS-A) and seven to symptoms of depression (HADS-D). Each item is scored from zero to three, with a maximum score of 21 for each scale. Frequencies of anxiety and depressive disorders were calculated using the patients' responses to the HADS items; the total score ranges from 0 to 21 points for each subscale. For HADS-A and HADS-D, the score ranges represent the severity of anxiety or depression: 0–7, no anxiety or depression; 8–10, mild anxiety or depression; 11–14, moderate anxiety or depression; 15–21, severe anxiety or depression (22). The HADS has been validated in Chinese population (23, 24).

### Statistics

Data were expressed as means ± standard deviations, medians (quantile: 1/4–3/4), or frequencies (percentages). All analyses were performed using IBM Statistical Package for the Social Sciences (SPSS Statistics 19.0, SPSS Inc., Chicago, USA). Parametric tests were used for continuous variables and non-parametric tests for ordinal, categorical or non-parametric variables. Differences between two groups of data were evaluated by paired *t*-tests or Wilcoxon matched-pairs signed-rank tests, when appropriate. Changes in normally-distributed variables over time were evaluated by one-way analysis of variance (ANOVA) with a repeated measures design, followed by Bonferroni's correction. The Chi-square test (or Fisher's exact test where appropriate) was used to analyse contingency tables and to compare proportions and/or frequency distributions. Multivariate logistic regression analysis was conducted to examine associations between demographic, clinical, physiological factors, and psychological symptoms, independently. All demographic and clinical factors were included in the multivariate analyses. *P* < 0.05 indicated statistically significant differences. All comparisons were two-tailed.

## Results

### Baseline Characteristics

**Figure 1:**
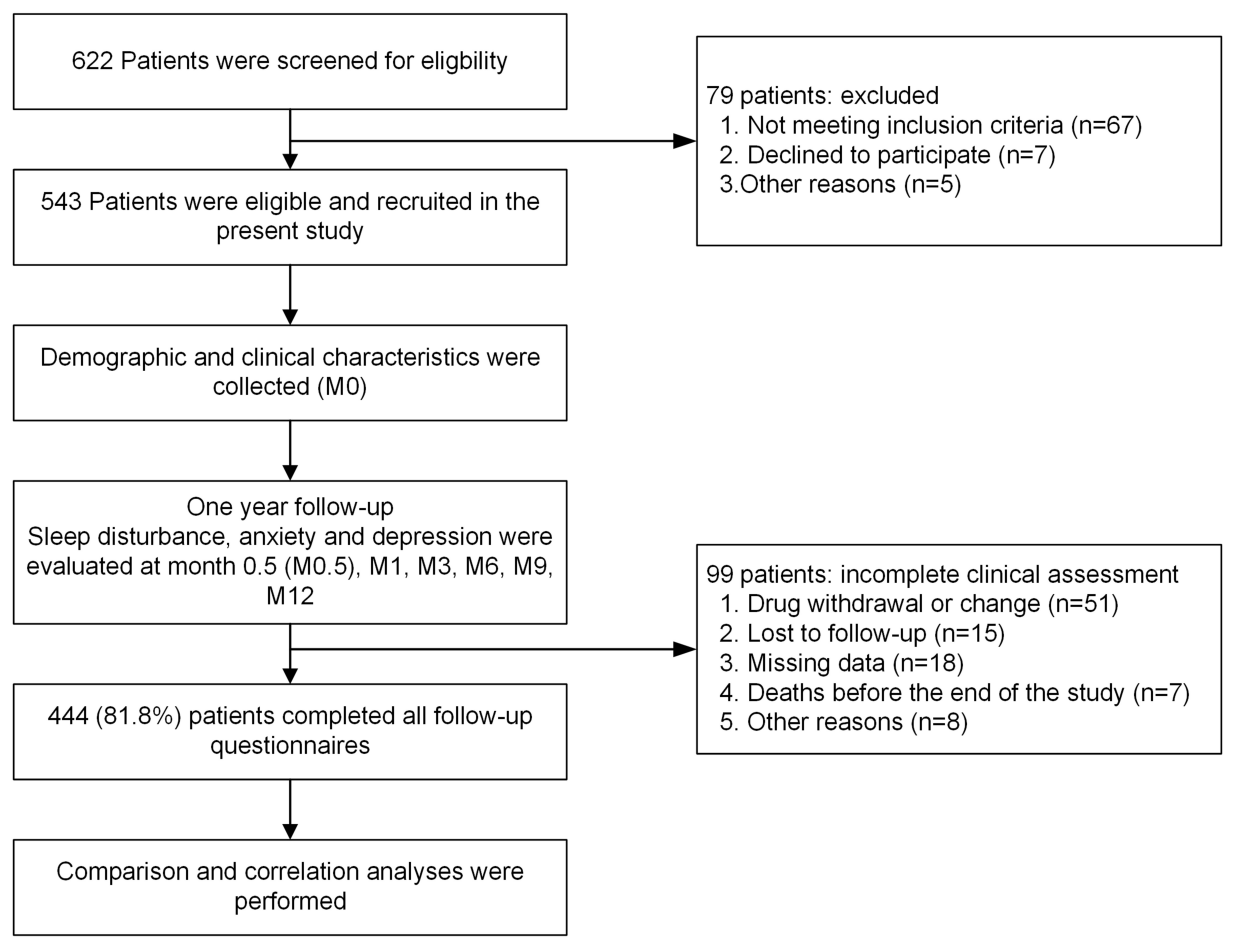
Flow chart of subject enrolment.

At baseline (M0), 543 patients with HIV infection were enrolled (Figure 1). The mean age was 35 years (*SD*: 11), 98.4% were male, and 91.9% were Han. The mean body mass index (BMI) was 22.5 kg/m^2^ (*SD*: 4.8), and 26.8% were obese. In terms of education levels, 356 (65.6%) patients had attained a college degree or higher, 125 (23.0%) were married, 40 (7.4%) were students, and 91 (16.8%) were unemployed. Ninety-seven (17.9%) patients began treatment within a week from HIV diagnosis, and 284 (52.3%) received treatment 1–4 weeks after HIV diagnosis. The mean CD4+ cell count at enrolment was 335 cells/mm^3^ (SD: 233), and 317 (58.4%) patients had CD4+ cell counts ranging from 200 to 499 cells/mm^3^. The mean HIV-1 ribonucleic acid (RNA) level was 19,175 (15) copies/mL. The other clinical characteristics, complications, and laboratory indices of all HIV patients are shown in Table 1.

**Table 1:** Demographic, clinical, and psychosocial characteristics of HIV patients.

**Characteristics**	**Category**	**Total participants**	
		***N* = 543**	**%**
Sex	Male	532	97.97
	Female	11	2.03
Age, years	<30	215	39.59
	30–50	264	48.62
	>50	64	11.79
Ethnicity	Han	492	90.61
	Others	51	9.39
Education	High school or lower	187	34.44
	College or higher	356	65.56
Marital status	Married	125	23.02
	Divorced/separated	39	7.18
	Single	379	69.80
Employment type	White-collar	223	41.07
	Blue-collar	189	34.81
	Student	40	7.37
	Unemployed	91	16.76
BMI	<18.5	53	9.76
	18.5–24	345	63.54
	24–28	108	19.89
	>28	37	6.81
Alcohol use in the past 3 months	No	398	73.30
	Yes	145	26.70
Cigarette use in the past 3 months	No	403	74.22
	Yes	140	25.78
Other chronic diseases	Hepatitis C coinfection	8	1.47
	Hepatitis B coinfection	33	6.08
	TB	4	0.74
	Diabetes	113	20.81
	Hypertension	98	18.05
Time since HIV diagnosis	<1 week	97	17.86
	1 week- 1 month	284	52.30
	>1 month	162	29.83
Sexual transmission route	Homosexual/bisexual	494	90.98
	Heterosexual	27	4.97
	Unknown	22	4.05
HIV-1 RNA (copies/mL)	<100,000	201	37.02
	10,000–50,000	169	31.12
	>500,000	173	31.86
CD4+ cell count, cells/μl	<50	37	6.81
	50–199	90	16.57
	200–499	317	58.38
	>500	99	18.23
WHO Stage	I	391	72.01
	II	112	20.63
	III/IV	40	7.37
cART initiated	TDF+3TC+EFV	497	91.53
	AZT+3TC+EFV	46	8.47

*HIV, human immunodeficiency virus; BMI, body mass index; TB, tuberculosis; RNA, ribonucleic acid; CD4+, cluster of differentiation 4-positive; WHO, World Health Organization; cART, combination antiretroviral therapy; TDF, tenofovir; 3TC, lamivudine; EFV, efavirenz; AZT, zidovudine*.

### Incidence of Drug Discontinuation

In this study, 444 patients (81.8%) completed all assessments during the 12-month follow-up and were included in the final analysis (Figure 1), while 99 were excluded due to lack of data throughout the whole 12-month follow-up period. For the 444 patients with complete data, a total of 51 (9.4%) patients discontinued treatment, 20 (3.7%) of which were due to EFV-related side effects, five (0.9%) were due to EFV resistance, and 26 (4.8%) were due to other causes. six (1.1%) discontinued due to NPAEs, 12 (2.2%) due to rash, one (0.2%) due to abnormal liver function, and one (0.2%) due to dyslipidaemia. In the six patients with NPAEs, two discontinued within the first 2 weeks, one at 1–2 months, two at 3–6 months, and one at 9–12 months after starting EFV treatment.

### Anxiety, Depression, and Sleep Disturbance at Baseline (M0)

The mean HADS-A and HADS-D scores of the 543 enrolled patients at M0 were 5.83 ± 4.13 and 4.94 ± 3.87, respectively. Of these, 165 (30.4%) patients had anxiety and 123 (22.7%) had depression at M0 based on the HADS-A and HADS-D scores. The mean PSQI score at M0 was 7.09 ± 3.08, and 370 (68.1%) patients were suffering from sleep disturbances.

Baseline anxiety, depression, and sleep quality scores for all 543 patients enrolled at the start of the study, the 444 patients who completed the 12-month follow-up, and the 51 patients with drug discontinuation are listed in Table 2. There were no significant differences in any M0 scores between any two of these three groups.

**Table 2:** Classification of the patients' anxiety, depression, and sleep quality at baseline.

**Parameter**	**Score**	**Participants enrolled at baseline**	**Participants completing the 12-month follow-up**	**Participants with drug discontinuation**
		**(*n* = 543)**	**(*n* = 444)**	**(*n* = 51)**
		**Frequency (%)**	**Frequency (%)**	**Frequency (%)**
Anxiety	Normal (0–7)	380 (70.0)	314 (70.7)	33 (64.7)
	Mild (8–10)	94 (17.3)	73 (16.4)	10 (19.6)
	Moderate (11–14)	47 (8.7)	40 (9.0)	5 (9.8)
	Severe (15–21)	22 (4.1)	17 (3.8)	2 (3.9)
Depression	Normal (0.7)	420 (77.4)	346 (77.9)	40 (78.4)
	Mild (8–10)	69 (12.7)	58 (13.1)	6 (11.8)
	Moderate (11–14)	38 (7.0)	29 (6.5)	4 (7.8)
	Severe (15–21)	16 (2.9)	11 (2.5)	1 (2.0)
Sleep quality	Normal (0–5)	173 (31.9)	138 (31.1)	13 (25.5)
	Poor sleep (6–10)	299 (55.1)	253 (56.9)	31 (60.8)
	Severely poor sleep (11–21)	71 (13.1)	53 (11.9)	7 (13.7)

*Anxiety, depression and sleep quality were assessed and categorized based on the Hospital Anxiety and Depression Scale (HADS-A, HADS-D) and the Pittsburgh Sleep Quality Index (PSQI), respectively*.

### HADS-D and HADS-A Scores Decreased Over the 12-Month Follow-Up

**Figure 2:**
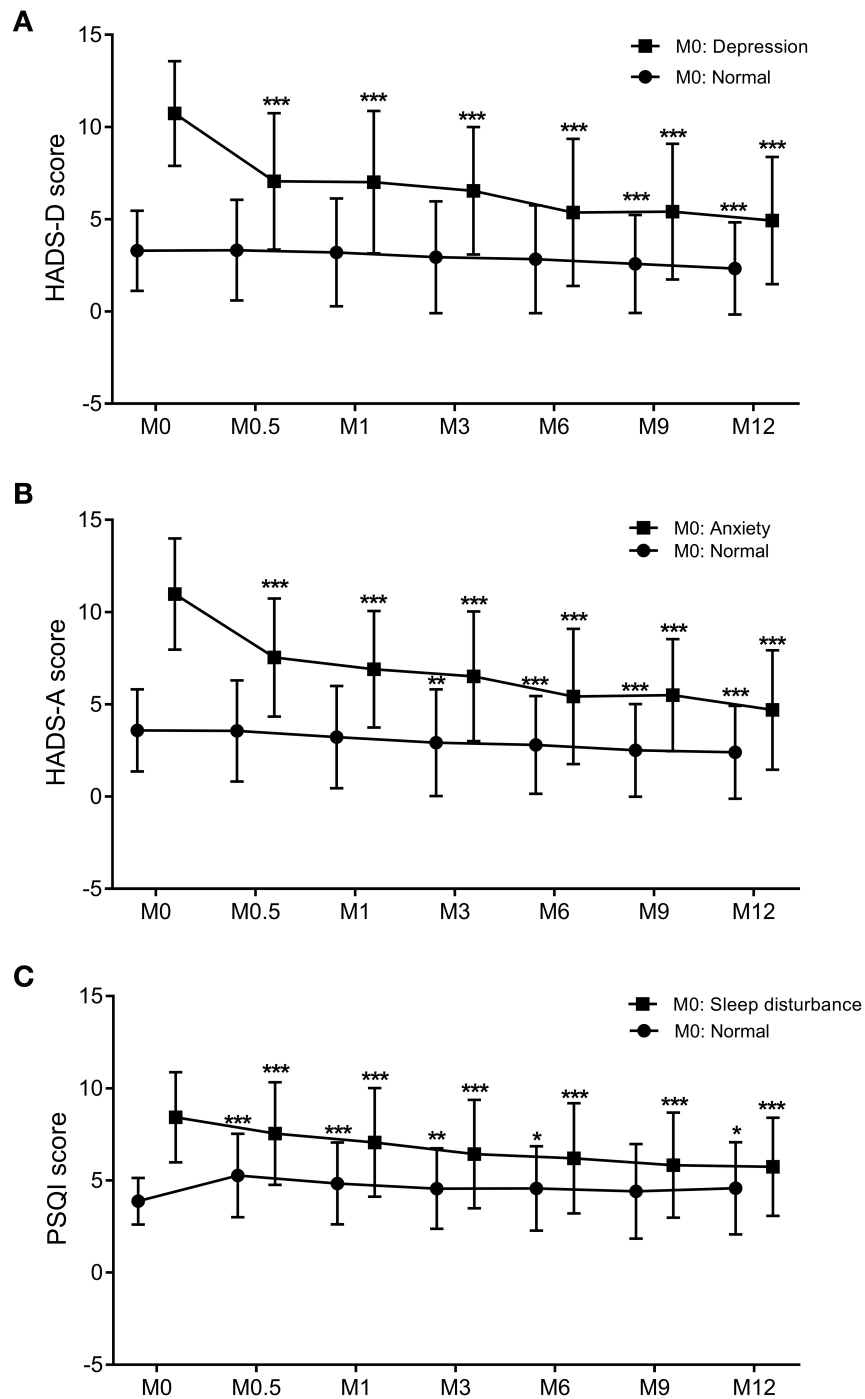
The trend of changes in the PSQI **(A)**, HADS-D **(B)**, and HADS-A **(C)** scores over the 12-months follow-up period. Graphs show the mean values and SDs at each trimester. Comparison between two groups was performed by paired *t-*tests. *p* < 0.05 was considered statistically significant. *: 0.01 < *p* < 0.05 compared to M0; **: 0.001 < *p* < 0.01 compared to M0; ***p < 0.001 compared to M0. PSQI, Pittsburgh Sleep Quality Index; HADS-D, Hospital Anxiety and Depression Scale-Depression subscale; HADS-A, Hospital Anxiety and Depression Scale-Anxiety subscale; M, month. Dots: individuals with normal HADS-A, HADS-D, or PSQI scores at baseline. Squares: individuals with abnormal HADS-A, HADS-D, or PSQI scores at baseline.

HADS-A and HADS-D scores steadily and significantly decreased at M0.5, M1, M3, M6, M9, and M12 compared to M0 baseline levels (Table 3, all *p* < 0.001). Based on HADS-A and HADS-D scores at M0, patients were stratified into normal M0-HADS-A (*n* = 314) and normal M0-HADS-D (*n* = 346) groups, and abnormal M0-HADS-A (*n* = 130), and abnormal M0-HADS-D (*n* = 98) groups. In both abnormal groups, the HADS-A and HADS-D scores significantly decreased at M0.5 compared to M0 (p <0.001); scores continued to decrease until M12 (Figure 2). In the normal group, the HADS-A and HADS-D scores were significantly lower compared to M0 from M3 (*p* < 0.01) and M9 (*p* < 0.001) onward, respectively, reaching their lowest values by M12 (Figures 2A,B).

**Table 3:** Mean differences (95% CI) and the significance of changes in HADS-A, HADS-D, and PSQI scores between each time point of follow-up vs. baseline (using paired *t*-test with missing values excluded pairwise).

	**HADS-A**			**HADS-D**			**PSQI**		
	**Mean diff**.	**95% CI**	***p*-value**	**Mean diff**.	**95% CI**	***p*-value**	**Mean diff**.	**95% CI**	***p-*value**
M0 vs. M0.5	1.03	0.53–1.53	<0.0001	0.79	0.30–1.27	<0.0001	0.17	−0.24 to 0.59	0.9061
M0 vs. M1	1.46	0.92–1.99	<0.0001	0.89	0.39–1.39	<0.0001	0.64	0.22–1.06	0.0002
M0 vs. M3	1.78	1.21–2.34	<0.0001	1.20	0.69–1.71	<0.0001	1.16	0.74–1.59	<0.0001
M0 vs. M6	2.19	1.61–2.76	<0.0001	1.54	0.98–2.10	<0.0001	1.32	0.84–1.79	<0.0001
M0 vs. M9	2.37	1.82–2.92	<0.0001	1.73	1.18–2.29	<0.0001	1.62	1.16–2.09	<0.0001
M0 vs. M12	2.69	2.08–3.29	<0.0001	2.03	1.49–2.56	<0.0001	1.64	1.17–2.10	<0.0001

*HADS-A, Hospital Anxiety and Depression Scale-Anxiety subscale; HADS-D, Hospital Anxiety and Depression Scale-Depression subscale; PSQI, Pittsburgh Sleep Quality Index; CI, confidence interval; M, month*.

**Figure 3:**
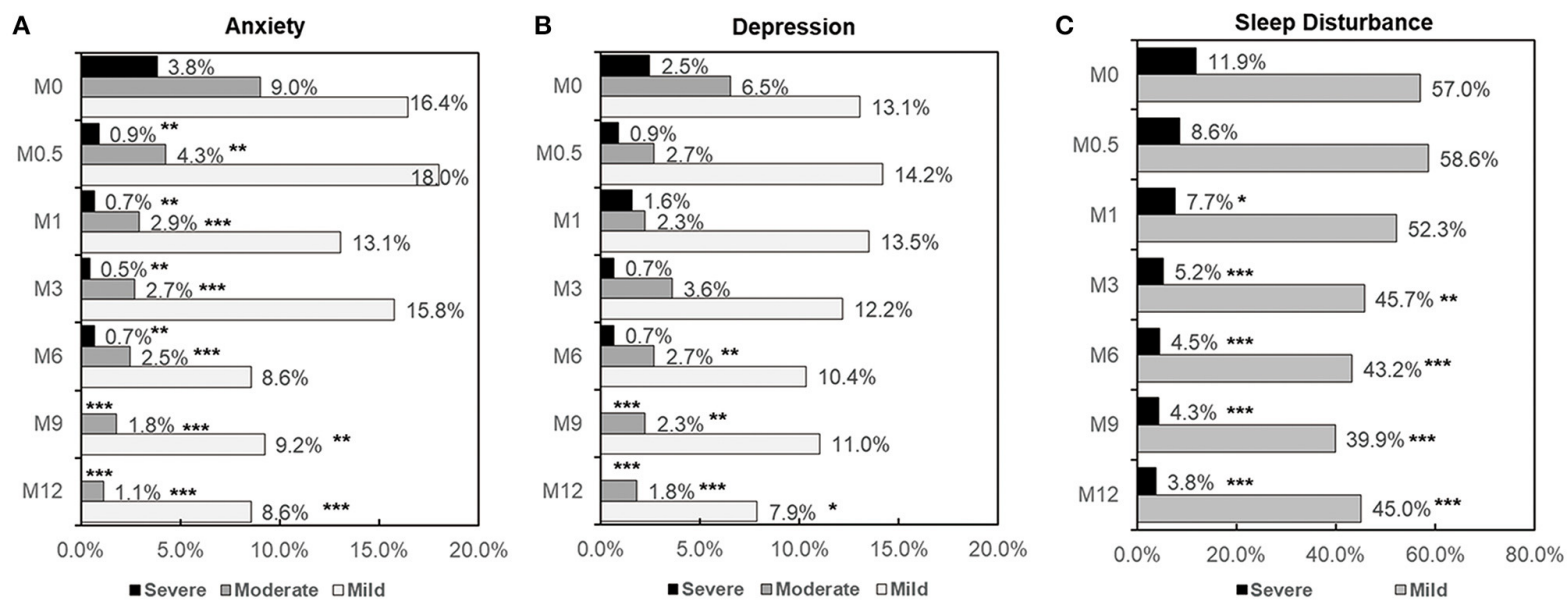
Frequency and severity of depression **(A)**, anxiety **(B)**, and sleep disturbance **(C)** at each time point in patients with HIV infection. Comparison of depression, anxiety and sleep disturbance rates between paired groups was performed by the McNemar test. Comparison of severity between two groups was performed by Chi-square test. *p* < 0.05 was considered significant. *: 0.01 < *p* < 0.05 compared to M0; **: 0.001 <*p* < 0.01 compared to M0; ***p < 0.001 compared to M0.

Based on the HADS-A and HADS-D scores, the frequencies of mild anxiety and depression at M0.5 were slightly higher compared to M0 (14.2 vs. 13.1% for HADS-D, and 18.0 vs. 16.4% for HADS-A, respectively), but the differences were not statistically significant. These frequencies decreased from M1 onward, reaching their lowest values at M12 (7.9% for HADS-D and 8.6% for HADS-A). For moderate and severe anxiety, the frequencies decreased significantly at M0.5, M1, M3, M6, M9, and M12 compared to M0 (Figure 3A, all *p* < 0.01). For moderate and severe depression, the frequencies significantly decreased at M6 and M9, respectively, compared to M0 (*p* < 0.01). No severe anxiety or depression scores were observed at M9 or M12 (Figures 3A,B).

### Sleep Quality Over the 12-Month Follow-Up

For all 444 patients, PSQI scores significantly decreased at M1, M3, M6, M9, and M12 compared to M0 (all *p* < 0.001, Table 3). Patients were stratified into two groups based on PSQI scores at M0 into a normal M0-PSQI (*n* = 138) and an abnormal M0-PSQI (*n* = 306) group. In the abnormal M0-PSQI group, the PSQI scores decreased significantly from M0.5 onward, reaching the lowest values at M12 (Figure 2C). However, in the normal M0-PSQI group, PSQI scores increased significantly at M0.5 compared to M0 (*p* < 0.0001, Figure 2C), then continually decreased from M1 to M12, although the PSQI score at M12 was still significantly higher than baseline M0 values (*p* < 0.05).

The frequency of mild sleep disturbance significantly decreased from M3 onward, reaching the lowest values at M12 (*p* < 0.0001). The rate of severe sleep disturbance significantly decreased at M1, M3, M6, M9, and M12 compared to M0 (Figure 3C).

### Risk Factors for Severe Anxiety, Depression, and Sleep Disturbances

To overcome the negative impact of NPAEs during EFV treatment, it is necessary to identify risk factors associated with severe anxiety, depression, and sleep disturbance. In our study, 89 (20.0%), 51 (11.5%), and 54 (12.2%) patients experienced at least one episode of severe anxiety, depression, and sleep disturbance at some point during the 12-month period. In order to assess predictive factors for these severe NPAEs, univariate logistic regression was performed (Table 4). High school education or lower (*p* = 0.04), being a student (*p* = 0.003) or unemployed (*p* = 0.001), and WHO stages of III/IV (*p* = 0.004) were associated with higher probability of severe anxiety. High school education or lower (*p* = 0.001), being a blue-collar worker (*p* = 0.02), unemployed (*p* < 0.001), or divorced/separated (*p* = 0.007) were associated with severe depression. A high school education or lower (*p* = 0.047), being unemployed (0.008), and having a viral load >50,000 copies/mL (*p* = 0.035) were associated with severely poor sleep. In addition, abnormal HADS-A, HADS-D, and PSQI scores at M0 were all associated with high probabilities of severe anxiety, depression, and sleep disturbance over the 12-month follow-up (Table 4).

**Table 4:** Univariate and multivariate logistic regression analyses to identify associations between demographic characteristics and severe anxiety, depression, and sleep disturbance over the 12-month follow-up period.

**Variable**	**Univariate**		**Multivariate**	
	**OR (95% CI)**	***p*-value**	**OR (95% CI)**	***p*-value**
**FACTORS FOR SEVERE ANXIETY**				
**Level of education**				
College or higher (reference)	1		1	
High school or lower	2.07 (1.03–4.15)	0.040	1.68 (0.69–4.07)	0.254
**Employment type**				
White-collar (reference)	1		1	
Blue-collar	1.98 (0.93–4.22)	0.076	2.13 (0.88–5.20)	0.095
Student	4.83 (1.71–13.62)	0.003	6.26 (1.93–20.31)	0.002
Unemployed	4.14 (1.85–9.28)	0.001	3.4 (1.29–8.92)	0.013
**WHO stage**				
I (reference)	1		1	
II	1.53 (0.77–3.07)	0.227	1.58 (0.73–3.45)	0.247
III/IV	3.73 (1.52–9.17)	0.004	4.39 (0.77–6.84)	0.007
Baseline HADS-A >7	5.72 (3.13–10.47)	<0.001	3.53 (1.69–7.39)	0.001
Baseline HADS-D >7	5.4 (2.98–9.78)	<0.001	2.71 (1.28–5.71)	0.009
Baseline PSQI >5	2.47 (1.17–5.21)	0.017	1.43 (0.62–3.31)	0.400
**FACTORS FOR SEVERE DEPRESSION**				
**Level of education**				
College or higher (reference)	1		1	
High school or lower	3.13 (1.57–6.28)	0.001	1.94 (0.80–4.73)	0.144
**Employment**				
White-collar (reference)	1		1	
Blue-collar	2.55 (1.16–5.63)	0.020	2.5 (0.94–6.63)	0.065
Student	2.11 (0.54–8.20)	0.280	2.58 (0.56–11.93)	0.224
Unemployed	5.87 (2.56–13.47)	<0.001	5.14 (1.85–14.29)	0.002
**Marital status**				
Single (reference)	1		1	
Married	1.87 (0.97–3.63)	0.062	1.28 (0.57–2.91)	0.552
Divorced/separated	3.71 (1.44–9.59)	0.007	3.89 (1.18–12.85)	0.026
Baseline HADS-A >7	5.59 (3.01–10.38)	<0.001	2.45 (1.13–5.30)	0.023
Baseline HADS-D >7	9.25 (4.91–17.42)	<0.001	5.08 (2.33–11.08)	<0.001
Baseline PSQI > 5	3.14 (1.38–7.17)	0.006	1.68 (0.66–4.27)	0.279
**FACTORS FOR SEVERE SLEEP DISTURBANCE**				
**Level of education**				
College or higher (reference)	1		1	
High school or lower	1.78 (0.98–3.24)	0.047	1.55 (0.78–3.08)	0.211
**Employment**				
White-collar (reference)	1		1	
Blue-collar	1.37 (0.79–2.39)	0.260	1.18 (0.61–2.26)	0.626
Student	1.48 (0.55–3.96)	0.438	1.69 (0.59–4.84)	0.328
Unemployed	2.38 (1.26–4.51)	0.008	1.73 (0.83–3.58)	0.143
**Viral load (copies/ml)**				
≤ 100,000 (reference)	1		1	
10,000–50,000	0.95 (0.52–1.75)	0.881	0.99 (0.52–1.89)	0.985
>50,000	1.81 (1.04–3.14)	0.035	1.72 (0.96–3.09)	0.071
Baseline HADS-A >7	2.4 (1.49–3.89)	<0.001	1.2 (0.67–2.14)	0.543
Baseline HADS-D >7	3.66 (2.21–6.06)	<0.001	2.69 (1.48–4.91)	0.001
Baseline PSQI >5	4.45 (2.23–8.90)	<0.001	3.49 (1.70–7.15)	0.001

*OR, odds ratio; CI, confidence interval; M12, month twelve; WHO, World Health Organization; HADS-A, Hospital Anxiety and Depression Scale- Anxiety subscale; HADS-D, Hospital Anxiety and Depression Scale- Depression subscale; PSQI, Pittsburgh Sleep Quality Index*.

All factors were included in the multivariate logistic regression analysis using the forward stepwise (conditional) method, which revealed that HADS-A (M0) >7 (*p* < 0.001), HADS-D (M0) >7 (*p* < 0.001), being a student (*p* = 0.002) or unemployed (*p* = 0.013), and WHO stages of III/IV (*p* = 0.007) were independent predictive factors for severe anxiety. Being unemployed (*p* = 0.002), divorced/separated (*p* = 0.026), and scores of HADS-A (M0) >7 and HADS-D (M0) >7 (*p* < 0.001) were predictive factors associated with severe depression. HADS-D (M0) >7 (*p* = 0.001) and PSQI (M0) >5 (*p* = 0.001) were independent predictive factors for severe sleep disturbances (Table 4).

### Risk Factors for Anxiety, Depression, and Sleep Disturbance 12 Months After EFV Treatment

As shown in Table 5, univariate and multivariate logistic regression analyses were performed to assess the risk factors associated with neuropsychiatric disorders at M12. Abnormal HADS-A, HADS-D, and PSQI scores at M0 were significant factors associated with anxiety, depression, and sleep disturbance at M12 based on univariate logistic analyses (Table 5). High school education or lower (*p* = 0.032), viral loads >50,000 copies/mL (*p* = 0.039), and WHO stages III/IV (*p* = 0.031) were associated with anxiety at M12. WHO stage III/IV (*p* = 0.018) was associated with depression and a high school education or lower (*p* = 0.036) was associated with sleep disturbances at M12. Multivariate analyses revealed that HADS-A (M0) >7 (*p* = 0.036), HADS-D(M0) >7 (*p* = 0.040), and PSQI (M0) >5 (*p* = 0.024) were independent factors associated with higher probability of anxiety, HADS-A (M0) >7 (*p* = 0.021) and HADS-D(M0) >7 (*p* = 0.001) were associated with depression, and HADS-A (M0) >7 (*p* < 0.001) was associated with higher risk of sleep disturbance at M12.

**Table 5:** Logistic regression analysis to identify associations between demographic characteristics and anxiety, depression, and sleep disturbance at M12.

**Variable**	**Univariate**		**Multivariate**	
	**OR (95% CI)**	***p*-value**	**OR (95% CI)**	***p*-value**
**FACTORS FOR ANXIETY AT M12**				
**Level of education**				
College or above (reference)	1		1	
High school or less	1.246 (0.583–2.662)	0.040	1.212 (0.522–2.815)	0.654
**Viral load (copies/ml)**				
≤ 100,000 (reference)	1		1	
10,000–50,000	0.594 (0.256–1.378)	0.225	0.783 (0.365–1.678)	0.529
>50,000	1.358 (0.676–4.727)	0.039	0.451 (0.180–1.127)	0.088
**WHO stage**				
I (reference)	1		1	
II	0.628 (0.254–1.552)	0.313	0.772 (0.291–2.047)	0.603
III/IV	1.957 (0.696–5.501)	0.031	0.801 (0.248–2.590)	0.711
Baseline HADS-A >7	4.311 (2.279–8.152)	<0.001	2.267 (1.055–4.871)	0.036
Baseline HADS-D >7	3.292 (1.740–6.231)	<0.001	2.273 (1.036–4.987)	0.040
Baseline PSQI >5	5.183 (1.818–14.774)	0.002	3.562 (1.182–10.737)	0.024
**FACTORS FOR DEPRESSION AT M12**				
**WHO stage**				
I (reference)	1		1	
II	0.768 (0.326–1.805)	0.545	0.686 (0.263–1.792)	0.442
III/IV	2.027 (0.719–5.709)	0.018	1.053 (0.314–3.539)	0.933
Baseline HADS-A	4.796 (2.521–9.125)	<0.001	2.526 (1.151–5.546)	0.021
Baseline HADS-D	6.215 (3.263–11.836)	<0.001	3.628 (1.652–7.968)	0.001
Baseline PSQI	2.654 (1.154–6.102)	0.022	1.351 (0.544–3.358)	0.517
**FACTORS FOR SLEEP DISTURBANCE AT M12**				
**Level of education**				
College or above (reference)	1	1		1
High school or less	1.578 (0.938–2.653)	0.036	1.594 (0.925–2.745)	0.093
Baseline HADS-A >7	2.864 (1.865–4.397)	<0.001	2.635 (1.603–4.333)	<0.001
Baseline HADS-D >7	1.800 (1.140–2.841)	0.012	1.056 (0.615–1.813)	0.843
Baseline PSQI >5	1.935 (1.283–2.920)	0.002	1.497 (0.965–2.322)	0.072

*OR, odds ratio; CI, confidence interval; M12, month twelve; WHO, World Health Organization; HADS-A, Hospital Anxiety and Depression Scale- Anxiety subscale; HADS-D, Hospital Anxiety and Depression Scale- Depression subscale; PSQI, Pittsburgh Sleep Quality Index*.

## Discussion

To our knowledge, this is the first prospective cohort study to evaluate the neuropsychiatric impact of initiating ART regimen containing EFV in HIV-infected patients in China. Our results demonstrated that: (1) in treatment-naïve HIV-infected patients, the EFV-containing regimen was associated with low risk of discontinuation due to NPAEs. (2) EFV-containing regimens was not associated with increased frequency of anxiety, depression, and sleep disturbance, and the HADS-A, HADS-D, and PSQI scores significantly decreased after 12 months of treatment. (3) Patients' baseline mental states and sociological factors including educational level and marital status were associated with severe neuropsychiatric disorders during the treatment period, and abnormal baseline HADS-A, HADS-D, and PSQI scores were risk factors for anxiety, depression, and sleep disturbance after 12 months of treatment.

HIV infection is a traumatic and stressful experience, and patients are more likely to exhibit mental health problems than the general population (25–27). Chronic HIV infection can adversely affect CNS function, leading to HIV-associated neurocognitive disorders (28). Among the enrolled patients in our study, there was a high rate of anxiety, depression, and sleep disturbance at baseline (30.4, 22.7, and 68.1%, respectively), consistent with the results of other studies in China (20, 29, 30). A systematic review in China revealed a relatively high prevalence of depression (>60%) and anxiety (>40%) in patients with HIV infection (31). This high rate of neuropsychiatric problems may be related to the psychological and social circumstances associated with HIV infection in China, including the psychological burden of HIV diagnosis, the aversive symptoms experienced by patients, the stigma and guilt of infection, the general prejudice and misconceptions about this disease (32), as well as the direct biological effects of viral infection (20, 33). Therefore, although we could screen for patients presenting with a history of confirmed neuropsychiatric disease, the underlying rates of neuropsychiatric disturbances before treatment still require further study.

We found no evidence that EFV-containing regimens resulted in increased frequency of anxiety, depression, and sleep disturbance after 12-months of treatment, consistent with several studies that demonstrated no significant differences in neuropsychiatric disorders in HIV patients receiving EFV compared to other ART regimens (8, 34–36). Although no apparent impact of EFV on neuropsychiatric symptoms was identified, we found that some patients could develop severe mental disorders during the 12-month treatment period. The subgroup with normal baseline HADS and PSQI scores showed an increased rate of anxiety, depression, and sleep disturbance at M0.5 (11, 12, and 13%, respectively), which may be related to the initiation of EFV treatment. These rates gradually decreased after M0.5, and there was almost no severe anxiety, depression, or sleep disorders at M12, confirming that the mental impact of EFV treatment is temporary and transient (6). Several studies have also demonstrated that the EFV-associated NPAEs do not increase the likelihood of discontinuation, as few patients need to stop treatment (8, 36). This is consistent with our study, as only six patients discontinued treatment due to NPAEs.

For patients who initiated ART with an EFV-containing regimen, there was a clinically meaningful improvement in neuropsychiatric symptoms based on HADS and PSQI scores over the 12-month follow-up period. Improvement in neurological performance after ART has not been observed in some of the studies concerning EFV treatment (11, 14). These differences may result from unique social and culture characteristics in China. The psychological improvement may due to timely and effective ART, as well as psychological intervention and education before treatment given by the clinicians in this hospital. The similar results have also been observed in two other studies (37–39). Successful treatments that reduced viral loads were associated with improvements in psychological distress levels (8, 35). Another possibility could be that the patients in our study were enrolled within a relatively short time interval following HIV diagnosis, with 70.0% of patients initiating ART <1 month after diagnosis. Although these patients may suffer from the psychological burden arising from the acute trauma of the diagnosis (31) as treatment is initiated, this problem is unavoidable, as the WHO recommends early and sustained HIV treatment regardless of CD4 count. Therefore, for these patients, effective ART use and healthy support systems may outweigh the increased probability of neuropsychiatric disorders caused by EFV-containing regimens. To some extent, the overall clinical benefits of treating patients with EFV could outweigh the risk of NPAEs. In order to analyse this reason, a long-term follow-up study in patients achieving viral suppression was needed to evaluate the neuropsychiatric changes after EFV treatment.

Several studies have highlighted the role of underlying neuropsychiatric disorders in predicting side effects of EFV (40–42). In our study, we found that baseline anxiety, depression, and sleep disorders were the most important predictors associated with NPAEs in patients treated with EFV. In addition, sociological factors such as education level, marital status, and employment status were also associated with severe psychological abnormalities during treatment, consistent with previous studies (43, 44). Since HIV/AIDS has become a chronic, manageable disease with the advent of highly effective ART, mental health management is required as an integral component effective of HIV/AIDS care (45). For patients in China, this kind of mental health education and support is often lacking; therefore, our findings suggest that in addition to assessing clinical characteristics, education and psychological support of HIV patients are also important and should be recognized by everyone.

There were several limitations to this study. First, the present study was a single-arm study, the absence of a comparator arm prevents us from drawing conclusions about the reasons for HADS and PSQI improvement after EFV treatment, and the findings would best be confirmed in a randomized clinical trial. Secondly, the patients were only recruited from a single metropolitan city (Beijing); therefore, the findings may not be generalizable to patients from all areas of China. Further studies recruiting more representative samples are needed to validate our results in Chinese populations. Thirdly, since the population recruited was predominantly male and participants with a homosexual or bisexual transmission route, our conclusions are only generalizable to people with the same characteristics. Fourthly, in the present study the risk factors of different clinical outcomes were analyzed by logistic regression analyses separately without multiple comparisons correction, thus the results were relatively preliminary and further evaluation is still needed. Finally, the psychiatric symptoms were evaluated based on patients' self-reported scores, so the severity of these symptoms may not be accurate, which could lead to reporting bias. However, based on the widely proved HADS and PSQI scale, the limit had been minimized and the results could strictly reflect the clinical relevance.

In conclusion, a low risk of drug discontinuation due to NAPEs was observed in treatment-naïve HIV-infected patients using EFV-based first-line antiretroviral therapy. The prevalence of severe anxiety, depression, and sleep disturbances was not increased after EFV treatment. Some key risk factors were identified that were associated with high probability of severe NPAEs over the 12-month treatment period. These factors could be useful for identifying patients with high-risk psychiatric disorders requiring a more in-depth evaluation and access to psychiatric health services along with HIV care and support.

## Data Availability Statement

The raw data supporting the conclusions of this article will be made available by the authors, without undue reservation.

## Ethics Statement

The studies involving human participants were reviewed and approved by the Clinical Research Ethics Committee of the Beijing Youan Hospital (LL-2019-038-K). The patients/participants provided their written informed consent to participate in this study.

## Author Contributions

WH, LS, ZL, SW, and LD conceived and designed the experiments. YS, YW, JY, BS, and TJ collected the sample information and contributed to reagents and materials. WH, SW, LD, QL, and AL performed the experiments. SW, LD, TZ, HW, SM, XW, and LS analyzed the data. SW, LD, and LS wrote the manuscript. All authors read and approved the final manuscript.

## Conflict of Interest

The authors declare that the research was conducted in the absence of any commercial or financial relationships that could be construed as a potential conflict of interest. The reviewers SS and JC declared a shared affiliation with the authors SM and SW respectively, to the handling editor at the time of review.
